# Exploring the Perspectives of Pediatric Health Care Providers, Youth Patients, and Caregivers on Machine Learning Suicide Risk Classification: Mixed Methods Study

**DOI:** 10.2196/57602

**Published:** 2025-08-19

**Authors:** Rohan R Dayal, Pua Lani Yang, Laura Nicole Sisson, Mira Bajaj, Shannon Archuleta, Sophie Yao, Daniel H Park, Hanae Fujii-Rios, Emily E Haroz

**Affiliations:** 1 Department of International Health, Bloomberg School of Public Health, Johns Hopkins University, 615 N Wolfe St, Baltimore, MD, 21205, United States, 1 9172837220; 2 Department of Health, Behavior and Society, Bloomberg School of Public Health, Johns Hopkins University, Baltimore, MD, United States; 3 Harvard Medical School, Mass General Brigham McLean, Boston, MA, United States; 4 Johns Hopkins University, Baltimore, MD, United States; 5 Department of Pediatrics, School of Medicine, Johns Hopkins University, Baltimore, MD, United States; 6 Department of Mental Health, Bloomberg School of Public Health, Johns Hopkins University, Baltimore, MD, United States

**Keywords:** suicide, electronic health records, adolescent, youth, clinician, machine learning, mixed methods, risk, human-centered, clinical practice, quantitative, joint display, EHR, clinical decision support, mental health, artificial intelligence

## Abstract

**Background:**

Suicide was the second leading cause of death for youth aged between 10 and 24 years in 2023, necessitating improved risk identification to better identify those in need of support. While machine learning (ML) applied to electronic health records shows promise in improving risk identification, further research on the perspectives of these tools is needed to better inform implementation strategies.

**Objective:**

These findings incorporate 2 studies aimed to explore patient, caregiver, and pediatric health care provider perspectives on suicide risk models and associated clinical practices. We sought to use these findings to inform the design and implementation of a suicide risk model and associated clinical workflow to improve the quality of care provided to at-risk youth.

**Methods:**

We conducted a convergent mixed methods study to evaluate pediatric provider perspectives by quantitatively surveying and qualitatively interviewing provider participants. The provider study was guided by the Consolidated Framework for Implementation Research, and data were analyzed descriptively and using a template analysis for quantitative and qualitative data, respectively. Qualitative interviews conducted among youth patients and caregivers as part of a sequential mixed methods study, guided by the Theoretical Framework of Acceptability, were analyzed using template analysis as well. The integration of quantitative and qualitative data was achieved through a joint display, and results were interpreted through a narrative review.

**Results:**

Forty-five providers completed the first section of the survey (risk model clinical preferences), while 44 completed the second section (risk model usability perspectives) and 38 completed the third section (Consolidated Framework for Implementation Research barriers and facilitators). Eight pediatric providers were interviewed. Ten semistructured qualitative interviews were conducted among 9 patient participants aged between 8 and 25 years and 1 caregiver. Overall, providers, patients, and caregivers expressed interest in applying ML methods to improve suicide risk identification. Providers felt that these tools could address current challenges in suicide-related care, such as inadequacies in manual suicide screeners or communication across care teams. Patients and caregivers saw potential for these tools to facilitate discussions regarding suicide and promote early intervention for patients who might otherwise be missed by current care practices. However, providers also expressed concerns about increased demand for mental health services, implications for patient confidentiality, coercive care, inaccurate diagnosis and response, and medical-legal liability. Ethical concerns shared by patients and caregivers spanned data safety practices and privacy regulations, respect for patient autonomy and informed consent, and decreased future health care engagement due to poor implementation.

**Conclusions:**

There is conditional acceptability and enthusiasm among providers, patients, and caregivers for ML-based suicide risk models. Successful implementation requires the incorporation of provider perspectives in a user-led fashion to build trust and empower clinicians to respond appropriately to risk flags, while upholding youth and caregiver perspectives to adequately accommodate patient needs.

## Introduction

Suicide was among the top 2 causes of death for youth aged 10-24 years in 2021 [1]. People who die by suicide often interact with the health care system in the year prior to death [2,3]. There is, therefore, an opportunity to identify those who may be at elevated risk of suicide and intervene to prevent suicide deaths [4-6]. Toward this end, health care facilities use various risk identification tools, such as clinical judgment, brief screening, and risk assessment processes [7]. Many screening tools have been shown to agree with measures of suicide ideation [8,9] and accurately identify individuals at risk in some studies [10-12], but the complexity of identifying suicide attempt or death risk remains a challenge for accurate risk identification [13]. Due to the complexity of identifying strong risk factors of suicide, there had been little to no improvement in the ability to predict suicide prior to the increased use of data-driven approaches [13].

In recent years, researchers have found success in accounting for the complexity of estimating suicide risk by applying machine learning (ML) to electronic health records (EHRs) to predict suicide-related outcomes due to their ability to test a combination of a wide variety of variables to identify the most accurate and generalizable predictive algorithm within a given dataset [13]. ML approaches to identify suicide attempt or death risk have even shown greater predictive ability than traditional approaches (ie, clinical assessments for suicidal ideation or capacity to attempt suicide), which surprisingly do not have greater predictive ability than chance alone [13]. For example, the ML model of Walsh et al [14] used more than 600 risk factors to produce accurate predictions for suicide attempts at varying time points for youth with and with no previous mental health outcomes. These results have been replicated by many other studies indicating high accuracy for ML models predicting future suicide ideation, attempt, and death [15]. While there are inherent limitations to any methods predicting rare outcomes such as suicide [16], ML is well suited to help make sense of and draw insights from health systems and other large data sources to help improve the identification of suicide risk.

However, despite advances in applying ML, there has been minimal research on how these tools can best be translated into clinical gain. To our knowledge, only a handful of studies around the implementation of risk models for suicide risk identification exist in the literature [17-24] and only 1 [25] has focused on implementation considerations for a youth (pediatric and adolescent) population. Formative research to explore the implementation of these kinds of tools can lead to more effective design and uptake [26]. The fields of implementation science and human-centered design offer robust methodologies that ensure that predictive analytics can be best leveraged for effective suicide prevention.

Previously, we found that an ML model developed using data from pediatric emergency visits at Johns Hopkins Pediatric Hospital improved the prediction accuracy of suicide risk within 3 months when combined with brief screening information [10]. In this study, we aimed to extend this work and explore the perspectives of pediatric providers, youth patients, and caregivers on the implementation of these types of suicide risk models and the functionality of clinical decision support tools using these models. As health care organizations consider the implementation of these models, understanding how they can best be used to inform care is critical to realizing their potential and preventing suicide.

## Methods

### Study Design

**Figure 1.**
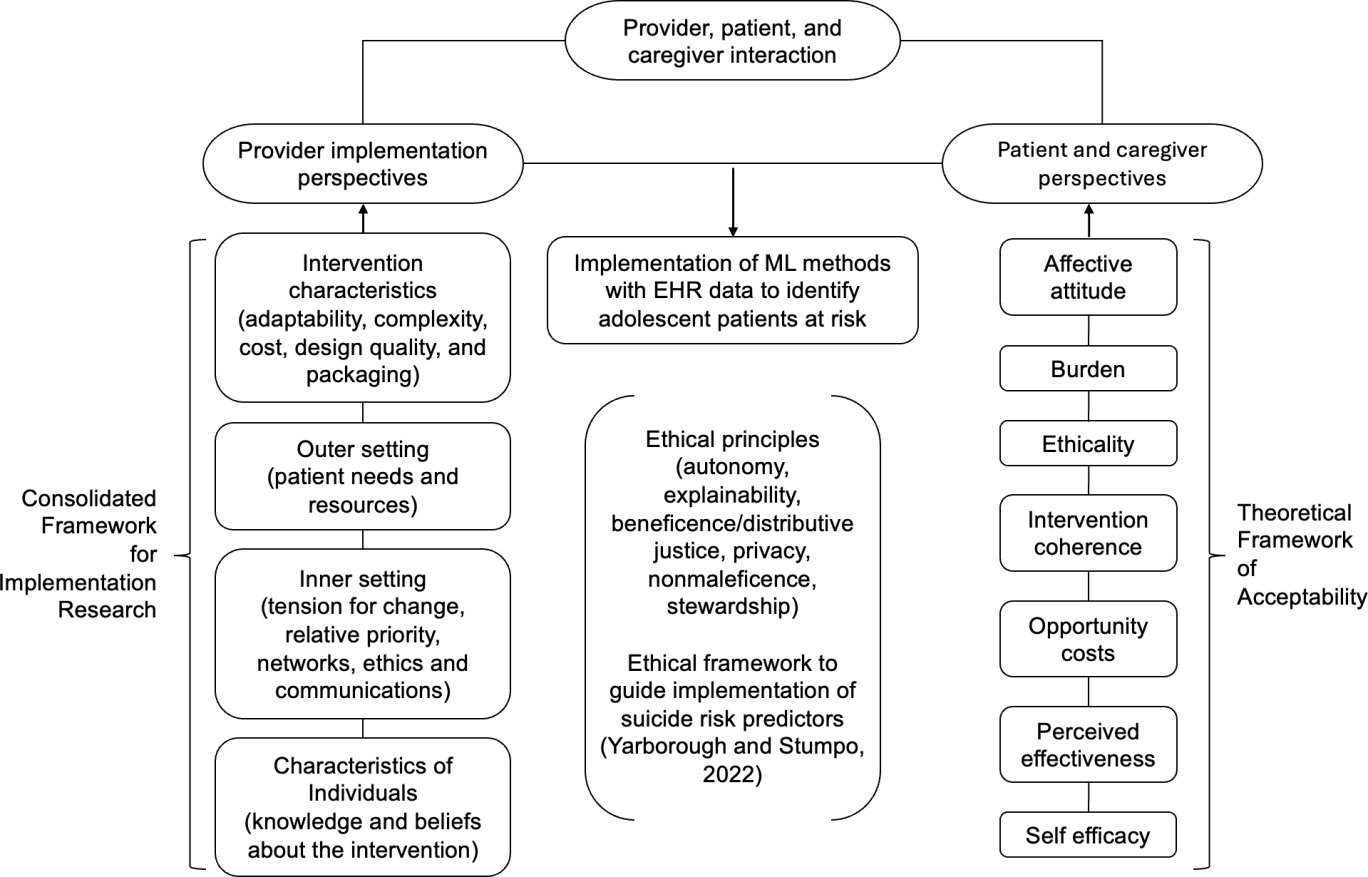
Conceptual framework for the integration of studies evaluating provider implementation perspectives and patient and caregiver perspectives on machine learning methods to identify suicide risk in youth. EHR: electronic health record; ML: machine learning.

This work incorporates 2 studies that were part of a larger effort to understand a wide range of stakeholders’ perspectives on predictive models for suicide prevention, incorporating constructs from 3 disparate but complementary frameworks (Figure 1). We used a concurrent mixed methods study design to evaluate pediatric provider perspectives and a sequential mixed methods design to evaluate patient and caregiver perspectives [27]. This manuscript incorporates quantitative and qualitative data gathered among providers and qualitative data gathered among patients and caregivers. Due to the length of the survey, quantitative findings of the patient and caregiver study will be reported in a future manuscript.

Relevant constructs for the provider study were drawn from the Consolidated Framework for Implementation Research (CFIR), a “metatheoretical” framework that synthesizes disparate implementation theories with a common taxonomy to facilitate theory-building of implementation science [28]. The Theoretical Framework of Acceptability (TFA) was incorporated into the framework of the patient and caregiver evaluation, as it aims to unify theories of acceptability to guide more effective, user-centered design of health care interventions [29]. Finally, an ethical framework for implementing suicide risk predictors was integrated to emphasize the need for patient-centered approaches to minimize the potential harms associated with automated risk identification [22]. Provider semistructured interviews were conducted over Zoom (Zoom Communications) between September and December 2022 and February and May 2025. Semistructured interviews among patients and caregivers were conducted over Zoom between May 2023 and March 2024.

### Study Settings

The study was conducted at Johns Hopkins Medicine and the Kennedy Krieger Institute. Johns Hopkins Medicine is a health care system based in Baltimore, Maryland, comprising 6 hospitals and more than 100 outpatient locations, serving more than 6 million patients annually. Kennedy Krieger Institute is a Johns Hopkins Medicine affiliate, providing care to 27,000 individuals with neurological, rehabilitative, and developmental needs annually.

### Participants

#### Pediatric Providers Participants

The study population included any ambulatory health care provider working in any care setting (including primary care, mental health, and emergency departments [EDs]) at Johns Hopkins Hospital (JHH) or Kennedy Krieger Institute who had provided care to a youth patient aged 10-24 years in the past. Our inclusion extended to 24-year-old patients, given the scope of this paper in focusing on pediatric provider perspectives, as there is no age requirement for pediatric care, and patients aged up to 24 years seek pediatric care on occasion. A convenience sampling approach was used to recruit providers. Recruitment emails with links to the survey and interview were disseminated to provider listservs in the child or adolescent psychology and emergency medicine departments by department administration. We aimed to survey up to 100 providers and interview 30 based on feasibility and the concept of saturation for qualitative research.

Survey participants had the option to schedule an interview at the end of the survey. Pediatric providers who did not respond to the listserv were also identified and recruited for the qualitative interview by email directly. There were no preexisting relationships between study participants and the study team.

#### Patients and Caregivers Participants

Adolescents and young adults aged between 12 and 24 years who participated in a previous study on suicide risk screening tools in the ED of JHH were recruited along with their caregivers to participate in this study. Additionally, we recruited ED patients older than 18 years who previously received services at the Kennedy Krieger Institute, JHH, or Johns Hopkins clinical practice networks using flyers and handouts. Adolescents recruited through the previous study on suicide risk-screening tools were excluded if they endorsed suicidality (assessed via the Columbia-Suicide Severity Rating Scale [30] or the Patient Health Questionnaire-9 [31]) throughout the 12-month enrollment period of the previous study. All patients and caregivers were excluded if they had a profound developmental delay or did not have a reading level at or above the eighth grade.

Patient and caregiver participants were given the option to complete a quantitative survey and optional qualitative interview or solely participate in the semistructured 1-hour qualitative interview after providing consent or assent. Youth and caregiver participants were recruited, interviewed, and compensated separately.

### Survey Instrument

The 19-item provider survey contained three sections that assessed the following aspects of ML-based suicide risk identification models for pediatric patients: (1) risk model clinical preferences, (2) risk model usability perspectives, and (3) CFIR potential barriers and facilitators.

The section assessing risk model clinical preferences began with a vignette that depicts a youth who presents to the ED and is classified by an ML algorithm to be at high risk for suicide (Multimedia Appendix 1). Following the vignette, 6 multiple-choice questions probed respondents’ preferences of when a provider should be notified of a patient’s risk, what the most important use of the risk flag is, and how it should fit into their existing workflow. Participants were also asked how much they would alter their care for a medium versus high-risk patient and how they would communicate risk status to patients. The section assessing risk model usability perspectives asked participants to rate 6 questions regarding tool acceptability using a 5-point Likert scale ranging from strongly disagree to strongly agree. Questions included opinions about the inclusion of the tool into their existing workflow, provider trust in the tool, and whether the tool can improve care for patients. Finally, the section assessing potential CFIR barriers and facilitators asked providers to label 7 statements related to the implementation context for the tool as a barrier, a facilitator, or neutral, and indicate whether the barrier or the facilitator will have a strong or weak effect on the ability to implement the tool. These 7 statements were developed using CFIR domains as a guide. Statements ranged from the cost to the health system in implementing the tool to whether physicians had ethical concerns about the use of such a tool.

In the survey and supporting vignette, inquiries on suicide risk did not specify a particular outcome (ie, suicide ideation, attempt, or death) to gain a more general perspective on a predictive tool rather than specific clinical implementation considerations. The provider survey was fielded through Qualtrics XM [32]. As advanced statistical methods were used in the patient and caregiver survey, including randomization to display differing vignettes to participants, the patient and caregiver quantitative survey was omitted from this manuscript and will instead be reported on in a future manuscript.

### Qualitative Interview Guide

#### Pediatric Provider Interview Guide

The provider interview guide was developed based on the CFIR and with additional input from previous qualitative studies on provider and patient perspectives of ML methods for suicide risk classification [24]. The final interview guide consisted of 21 questions that are separated into three parts: (1) exploratory inquiry, (2) reactions to a suicide risk classification prototype, and (3) perspectives on implementation (Multimedia Appendix 1). The exploratory inquiry section included 4 prompts about suicide care, including knowledge about existing programs, screening methods, and roles. The reactions to a suicide risk classification prototype section first described a basic three-step process of how the implementation of this tool could look accompanied by a visual mock-up: (1) patient data are captured in their EHR, (2) the suicide risk score is passively calculated based on variables included in each patient’s EHR, and (3) if the patient is considered to be at risk for suicide, a risk flag for further risk assessment populates on the patient profile screen alerting the provider before the visit. Following this description, we asked the participants 12 prompts about their opinions on the tool, including their perception of its complexity and potential integration. The last section, perspectives on implementation, asked participants about CFIR-informed barriers and facilitators to implementation in their care setting. The final guide was checked for relevance prior to its use by 2 providers who fit the inclusion criteria for this study. Their feedback was provided through email and then was integrated into the final version. Similar to the quantitative survey, inquiries on suicide risk did not specify a particular outcome (ie, suicide ideation, attempt, or death) to better assess general perspectives toward suicide risk identification tools.

#### Patient and Caregiver Interview Guide

The youth patient and caregiver interview broadly inquired about participant thoughts on clinical practices physicians use to assess suicide risk, how physicians could better support patients and their families, and implementation considerations of ML-based tools that predict suicide risk (Multimedia Appendix 1). First, patients and caregiver participants were probed on attitudes toward ML and its use in health care settings. Participants were then read a short vignette about an adolescent at a non–suicide-related medical appointment who was unknowingly identified as being at high risk for suicide by a new ML-based tool. Participants were probed on their attitudes toward the vignette scenario, care preferences for the hypothetical patient, and attitudes toward the use of personal health data to identify suicide risk. Questions in the interview assessed participant perspectives on whether such tools should be used, consent for the usage of such tools, the use of demographic information for these tools, and how physicians should attempt to inform patients about these tools.

### Data Analysis

Quantitative results for the provider survey were descriptively analyzed using Qualtrics XM (version November 2022) [32] and Microsoft Excel. Those who consented to participate but did not specify their occupation to answer any questions were omitted from analysis. All qualitative data were analyzed in Dedoose Desktop (version 9.0.17) [33]. A codebook approach to qualitative thematic analysis was used for both the provider and patient or caregiver interviews, applying the CFIR and TFA as the guiding frameworks to data collection and thematic structure, respectively [34]. We used template analysis, a method developed by King et al, to develop a final hierarchical coding structure to apply to our interview data [35,36]. First, the research team familiarized themselves with the interview data, writing analytic memos after conducting each interview and during the transcription process to reflect and synthesize musings. Preliminary coding was done by 2 coders, who inductively coded the first interview and reviewed the application of codes with the entire research team. Inductive codes were evaluated in the context of the raw data, deductive coding frameworks of the CFIR for provider interviews and TFA for patient or caregiver interviews, and memos to develop a final codebook that organized inductive codes within the CFIR and the TFA. This initial coding template was then applied to the rest of the transcripts, which were evenly distributed between 2 coders of the research team (PLY, LNS, SY, HYP, and RRD), with additional inductive codes added when emergent themes were not captured by the existing codebook.

#### Pediatric Provider Codebook Analysis

The preliminary provider interview codebook contained 21 codes agreed upon by both coders (PLY and LNS), covering topics such as patient autonomy or risk flag usefulness. A total of 35 inductive codes were organized under the following CFIR domains and subdomains: intervention characteristics (subdomains: adaptability, complexity, and design equity and packaging), outer setting (subdomain: patient need and resources), inner setting (subdomains: tension for change and networks), and characteristics of individuals (subdomains: knowledge and beliefs about the intervention and ethical considerations). Four additional inductive codes were not characterized under the CFIR framework: (1) patient-provider communication, (2) implementation barriers, (3) high-risk patients, and (4) stigma related to mental health. Finally, integrative themes [36], or those that permeated across several codes in the CFIR, were used to conceptualize the quantitative data through a joint display.

#### Patient and Caregiver Codebook

The preliminary patient and caregiver codebook contained 23 inductive codes agreed upon by both coders (PLY and SY) aimed to capture themes for youth and caregiver preferences on implementation and suggestions for clinical practices. The final codebook included 25 inductive codes organized by the 7 following deductive TFA constructs: affective attitudes, burden, perceived effectiveness, ethicality, intervention coherence, opportunity costs, and self-efficacy. Two additional inductive constructs with 16 total subcodes were added to capture preferences or suggestions to improve suicide-related care for youth (construct: care preferences) and the personal, community, or structural environment characteristics that may impact implementation (construct: implementation environment). Constructs were removed or redefined due to theme similarities to result in the final integration of themes in the joint display.

### Mixed Methods Integration

Mixed methods integration occurred during sampling, data analysis, and final interpretation of study findings [37]. A joint display indexed by the CFIR and TFA conceptual frameworks was used to represent mixed methods provider results and patient and caregiver qualitative findings through visual means to facilitate meaningful metainference of the data [38,39]. The results were then interpreted through a narrative review and augmented by prominent qualitative themes not captured by the display.

### Ethical Considerations

The studies assessing provider and patient or caregiver perspectives on ML implementation in suicide-related care were approved by the institutional review board (IRB) of Johns Hopkins Bloomberg School of Public Health (IRB number 22521 and IRB number 00042872, respectively). Provider participants gave informed consent electronically prior to study participation. Adolescents younger than 18 years provided assent, and youth older than 18 years and caregivers provided informed consent for participation after taking a 4-question comprehension assessment and having any of their questions about the terms of the study answered by the study team. Consent was recorded in the form of a checkbox question for those who completed the surveys, or orally if participants elected to participate only in the qualitative interview. All qualitative data were deidentified during the transcription process to ensure that no identifiers remained in any quotes reported. Similarly, all identifiers were removed from the provider quantitative data upon completion of the study. Provider participants who completed the quantitative survey were provided the opportunity to enter a raffle for 1 of 3 US $50 Amazon electronic gift cards, and patient or caregiver participants were compensated with US $5 Amazon electronic gift cards for completing the survey. All interview participants were compensated with US $30 Amazon electronic gift cards for completing the interview.

## Results

### Study Participants

The description of study participants can be found in Table 1. Of the 76 health care providers who viewed the survey, 73 providers were eligible, 61 consented to participate, 45 answered at least 1 question and completed the first section of the survey, 44 completed at least 1 question in section 2 of the survey, and 38 providers completed at least 1 question in section 3 of the survey. The average duration to complete the survey among those who completed at least 1 question in section 3 of the survey was approximately 17 minutes. Out of those who consented and answered at least 1 question but did not complete the survey (n=24), most (n=16) stopped after the first question. The average duration of time spent on the survey was approximately 112 minutes. To reduce potential bias, we reported all survey item results within the sample of 45 providers who completed at least 1 question.

**Table 1.** Occupational characteristics of pediatric provider survey and interview participants.

Characteristics	Survey participants		Qualitative interview participants
	Consented sample (n=61), n (%)	Completed at least 1 question (n=45), n (%)^a^	Sample (n=8), n (%)
Occupation			
Psychologist	30 (49)	23 (51)	2 (25)
Psychiatrist	N/A^b^	N/A	2 (25)
Other physician^c^	22 (36)	16 (36)	3 (38)
Social worker	4 (6)	3 (7)	N/A
Nurse practitioner	3 (5)	2 (4)	1 (13)
Nurse	1 (2)	1 (2)	N/A
Other	1 (2)	N/A	N/A

a All participants who completed at least 1 question in the quantitative survey also completed a majority of questions in section 1 of the quantitative survey, which included questions assessing the clinical preferences of providers in implementing a potential risk model.

b Not applicable.

c The “other physician” category included those who specialized in primary care, emergency medicine, or other subspecialties.

For providers, among those who consented to participate in the quantitative survey and completed at least 1 question, 23 (45, 51%) were psychologists, 16 (45, 36%) identified as physicians, 3 (45, 7%) were social workers, 2 (45, 4%) were nurse practitioners, and 1 (45, 2%) was a nurse. Of the 8 qualitative interview participants, there were 2 psychologists, 2 psychiatrists, 2 primary care physicians, 1 pediatric neurologist, and 1 nurse practitioner (Table 1). Three of the provider interview participants were recruited for the interview directly and did not participate in the quantitative survey. Responses to each survey item among providers who consented and completed at least 1 survey question are included in Tables S1-S3 in Multimedia Appendix 2. Out of 22 patient and caregiver participants who expressed interest in completing a qualitative interview, 9 youth patients and 1 caregiver elected to participate in the optional qualitative interview. Youth interviewees had a mean age of 22.8 (SD 0.8) years, and patient and caregiver interviewees were primarily non-White identifying (White: n=1; Black or African American: n=2; and Other/Prefer not to answer: n=6). The metainferences resulting from the combination of quantitative results among providers and qualitative themes among providers, patients, and caregivers are included in the joint display (Multimedia Appendix 3). Results are provided stratified by CFIR domains and the metainferences narratively summarized in the following subsections.

### Intervention Characteristics: Adaptability, Design Quality and Packaging, and Cost

The majority (28/45, 62%) of survey respondents, regardless of occupation, preferred a risk flag that alerts providers to imminent risk that is shown in advance of a patient visit as opposed to an alert for risk that may be more chronic and indicate risk over the next 6 months, or year. Interviewees preferred the risk alert user interface to be prominent (in contrast to the small yellow flag on the prototype) and desired seamless population of the risk alert information into the patient’s note. Thoughts on the trade-off between sensitivity and specificity of the risk alert varied across participants of different provider types. For example, 1 pediatrician preferred a more specific risk alert but cautioned that there is a limit: “If you get too many false positives, people will ignore it. I would like it to have more false positives than false negatives... Because you want something like that to give you the sense-It may be a false sense of security, but the sense that you’re not sending too few.”

To support the uptake and use of the tool, adequate training to ensure accurate interpretation of and response to the risk flag was desired. Interviewees perceived a need for enhanced decision support tools, such as recommended next steps (eg, referrals), additional manual screenings, and safety planning, to offer clear protocols that would benefit especially nonmental health providers. As 1 primary care provider shared, “For those of us who are your run-of-the-mill providers, we’re not asking those questions every day and fortunately, it doesn’t happen that often. But when it does, we want to have that next screening tool available to us.” Fears of inadequate implementation support led to providers describing “decision paralysis,” or the inability to make a treatment decision in difficult scenarios, such as those where a patient is identified but does not present to their visit, or if high-risk patients deny follow-up care. Although survey respondents did not identify the financial cost to the health system as a barrier to implementation, interviewees anticipated that the tool may increase demand for mental health care.

Youth and caregiver concerns for misdiagnosis highlighted a difference in perspective with providers regarding imminent risk flags. Youth participants believed that misclassification may be common in identifying imminent risk due to artificial intelligence’s (AI’s) potential inability to account for imminent risk factors that are dependent on situational and personal factors, as these are not recorded in the EHR. One participant stated, “There’s just so many things that the AI wouldn’t exactly know, other than the visits and things that are usually written in medical records, like personal stuff.” However, these participants also approved of implementing an ML tool due to their ability to improve the allocation of early intervention resources to patients experiencing mental health crises. This advantage was perceived to be especially important in scenarios where patients hesitate to bring up these concerns with their providers voluntarily. For example, one participant stated, “This tool can [be] another way that the person can get the care that they need that they might be either too afraid to ask about or that they aren’t aware that they need.”

### Inner Setting: Tension for Change and Networks

There was acceptance of predictive analytic methods and their potential to improve current suicide prevention practices in clinical settings. Nearly half of all survey respondents (18/45, 40%) felt that these kinds of approaches are needed, and about half (23/45, 51%) felt that they aligned with leadership goals. Interestingly, less than half of the psychologists (10/23, 43%) surveyed perceived a need for more prevention approaches. Interviewees agreed that these kinds of approaches could be useful for making informed care decisions and could potentially address current challenges to identifying patients at risk of suicide. For example, manual screening tools designed to assess current risk were described as burdensome for providers to administer. There are also concerns about limitations in their use, as 1 interviewee who sees patients with cerebral palsy commented, “I would say probably sixty to seventy percent of my patients have some sort of cognitive delay. So those parents are kind of like, what is the point in asking these questions?” Notably, all interviewees viewed ML methods positively, if implemented well. As 1 interviewee commented on the helpfulness of a ML tool, “I think it really depends on how it’s done and how it’s implemented….I think we absolutely need to find those kids that are at risk.”

Approximately half (24/45, 53%) of the survey respondents, and a majority of psychologists (13/23, 57%), indicated that designing these methods to improve communication across care teams would help facilitate their implementation. Interviewees corroborated this, highlighting communication with other providers as critical to making decisions for patient care as well as balancing internal caseloads. For example, when asked the extent to which viewing other providers’ notes is helpful, 1 interviewee responded: “Very, very helpful and informative. That really saves a lot of time there. Even what’s said in the provider’s note and what is understood by the patient, or the family is sometimes very different. So I kind of need to know, what was the plan of care discussed with the family? What did the provider want versus what is actually happening?”

Youth and caregivers noted current limitations in suicide-related care as well. Time constraints related to their visit were often discussed as reasons for patients not disclosing mental health concerns with their provider. One participant described their frustrating experience of trying to initiate a discussion with their provider about mental health: “I always feel like, I’m gonna talk to my doctor about this. Like it’s a very big concern and then when I get there, it’s just like so chaotic, and they’re just like rushing in and out. And I just feel like I can’t actually talk to them.” Participants also mentioned the difficulty in disclosing mental health concerns during a non–mental health–related visit, or with a new provider. When recounting their personal experience, one participant stated, “It’s really hard for me to actually talk about it with someone. Unless I know them….If it’s just like a random provider, I think it’d be a lot harder to answer questions like verbally.” These limitations contrasted with the perceived potential of ML to prompt providers to assess mental health and suicide risk during visits, better prioritizing mental health discussions especially among patients who might not voluntarily raise these concerns.

### Outer Setting: Patient Needs and Resources

On the provider side, participants generally agree that communication of ML-determined risk status requires close collaboration with patients and their families while carefully navigating the interplay of confidentiality, stigma, and youth-caregiver relationships. Nearly half of all survey respondents (18/45, 40%) preferred to discuss a patient’s risk status with them during an upcoming visit. Interviewees stressed the importance of having a trusting relationship with their patient to inform how they might approach the conversation, anticipating that their patients’ reactions to this information could vary by their level of engagement and care histories. Cultural sensitivity and responsiveness were a key consideration, including the impact of cultural values and social norms on mental health and the sequelae of inpatient admission for patients who are racially minoritized. Not having providers on a care team who share cultural identities and backgrounds with patients was identified as a major limitation to the availability of culturally responsive care.

With an adolescent population, engagement of caregivers was another critical consideration, including the youth-caregiver relationship, caregivers’ level of engagement in care, and the patient’s age and cognitive ability. Interviewees anticipated challenges such as youth not wanting to inform their caregivers about their risk status or caregivers’ negative perceptions of mental health care. Providers working with patients with disabilities anticipated that caregivers would be generally receptive to the tool but navigating patient confidentiality issues made it more complex, given the caregivers’ involvement in their youth’s daily functions. One interviewee shared an example of a patient not wanting to have their parent know of their risk, and that “some of them are not going to want their family members who kind of have their hand in every other aspect of their lives, helping them bathe, helping them dress, helping them do everything else, so they don’t have control over a lot of other aspects of their life.” In such cases, providers must balance advocating for their patient’s needs while upholding their professional obligation to protect the confidentiality of vulnerable patients.

Youth and caregiver perspectives emphasized the importance of sensitive and tailored communication by providers to build trust, prioritize patient autonomy, and better balance efforts to treat a given patient’s chief complaint and assess for suicide risk. Interviewees felt that overriding one’s decision to disclose mental health concerns during a visit can discourage future health care engagement. One interviewee exemplified this by stating, “If I twist my ankle and a doctor uses all the time to ask about my depression instead of treating my ankle, it’d feel like the doctor’s not listening to my problems.” Interviewees also emphasized the importance of assessing readiness to engage in mental health discussions among patients and their families. This was especially important for interviewees in discussing adolescent patient communication needs. For example, one participant suggested that implementation efforts should train providers to assess familial mental health perceptions to “guide how the provider approaches the conversation because you don’t want to do something that’s going to put the patient kind of in an unsafe situation.”

### Characteristics of Individuals: Knowledge and Beliefs About the Intervention (Ethicality)

Almost half of survey respondents (22/45, 49%) and all interviewees raised concerns about the ethics of predictive analytics in identifying risk of suicide. Interestingly, more psychologists (14/23, 61%) viewed the potential ethical implications of predictive analytics as a barrier than physicians (7/16, 44%), while a greater percentage of physicians (5/16, 31%) than psychologists (5/23, 22%) were neutral toward the ethical implications of predictive analytics. Interviewees stressed that sufficient quality, accessible, and culturally appropriate mental health care resources must be available to act on the risk flag before it should be implemented. Other ethical concerns involved the consequences of inaccurately classifying a patient’s level of risk, inadequate expertise or training of the clinician to interpret the flag and respond appropriately, use of population-level or irrelevant data as model inputs, potential to negatively impact quality of care, compromising patient confidentiality, and care standards in cases where high-risk patients do not present to their visit. Potential liability issues were of concern, as one interviewee shared, “I worry that we’ll be responsible for people that aren’t right in front of us. And that will definitely be hard. It will put an extra burden on the provider to be in touch with them, and then what happens when they can’t be in touch with them? It just sets up a whole cascade of the balance of risk and what to do.” Furthermore, additional scrutiny of the accuracy and data inputs of ML methods is needed specifically for patients who have been racially and systematically minoritized and for patients with developmental disabilities. Interviewees desired more concrete information about how a patient’s history and context are involved in their risk classification to ensure that they are being fairly and equitably identified. Giving patients the ability to opt in or out of the risk model, select who has access to their data, and self-administer assessment tools were all suggested as ways to actively involve patients in their risk determination and care.

Youth and caregiver participants expressed skepticism that current security measures were sufficient to protect their data and shared concerns for potential breaches of confidentiality. Some participants questioned whether current regulations and patient privacy protections were well equipped to handle increased technological integration without leaving patients vulnerable to exploitation by health systems or private organizations. For example, one interviewee questioned whether companies may have the ability to purchase private health information, questioning, “How secure is AI?…Can people, like, access it? Can companies buy that data?” However, others stated that ethical approval of these tools by patients was contingent on reassurance by providers that privacy and Health Insurance Portability and Accountability Act protections were equipped to handle the implementation of these models. Similar to providers, youth and caregiver participants felt that obtaining explicit consent for using personal health information was imperative. Potential patients and their families need to be counseled on how their information is collected, stored, and used to better provide informed consent for the use of their personal information in AI-based tools. One interviewee shared that using ML tools to guide treatment without explicitly providing consent could “go against the patient’s will for that type of treatment.” Ensuring informed consent and establishing transparency regarding data use and storage practices were key to gaining youth and caregiver support of implementing AI-based suicide risk tools.

## Discussion

### Principal Results

This study aimed to explore the perspectives of pediatric providers, adolescent and young adult patients, and caregivers on the implementation of suicide risk models and preferences regarding clinical decision support functionality. There is overall enthusiasm for and interest in using predictive analytics to improve the early identification of suicide risk among pediatric health care providers, youth patients, and caregivers in clinical settings. Providers indicated that effective and ethical implementation should include transparency in the development of the ML algorithm, allocation of corresponding resources to address patient needs, medical-legal liability, and preferences for a user-friendly interface integrated into existing workflows, as well as enhanced resources for clinical decision support. Youth patients and caregivers also recognized the limitations of these tools, highlighting the importance of engaging in conversations with health care providers about mental health status to account for the temporality in suicide risk to better generate an accurate depiction of individual risk.

Our findings suggest that a predictive analytic tool for suicide risk should inform providers of patients who are at imminent risk prior to an upcoming visit. The alert itself should be attention-grabbing and consistent with the current EHR user interface. Interaction with the alert should be intuitive and easy, and providers should be able to see the contributing factors to a patient’s risk score. Having recommended next steps to accompany the risk flag would be beneficial for all providers but more so for those who see high-risk patients less often. Given preferences to use the tool in combination with manual screenings, clinical decision support might provide guidance for appropriate screening and risk assessment questions to be administered during the upcoming visit. Providers should receive training to interpret the risk flag accurately and implement next steps in a culturally informed manner.

Interviews with youth patients and caregivers stressed the importance of respecting patient autonomy and the need to enforce stronger data privacy protections. With youth populations, trainings should include navigating the limitations of confidentiality with the patient and communicating risk status to caregivers. Training might draw from the experiences of mental health providers who have navigated difficult conversations with caregivers and have communicated with them through patient notes.

### Comparison With Prior Work

Implementation preferences identified through this work align with qualitative research conducted with providers who work with adult populations. Providers in these settings voiced similar barriers and concerns, including the quality of model inputs, liability implications, alert desensitization, increasing demand for mental health care without ensuring availability of care, and stigma [17,23,40]. In addition, providing adequate training, clear protocols to respond to alerts, and clinical decision support were recommended [17,40]. Overall, providers were generally supportive of predictive models with demonstrable added value and accuracy [17]. Patients and caregivers in this sample expressed similar concerns regarding data safety practices to those found by Davis et al [25], with additional considerations for fears of data being accessed by organizations or groups outside of the health system. This research extends these findings to implementation with a youth population that requires a nuanced approach toward patient confidentiality and youth-caregiver relationships.

Implementation preferences identified in this study also align with perspectives of pediatric providers toward suicide risk models. These providers similarly highlighted the importance of cultural sensitivity in communication efforts, as well as the importance of establishing connections between risk models and mental health care resources [25]. The qualitative findings of Davis et al [25] extend our recommendation for provider training to respect confidentiality among youth patients, stating that this can be done by delivering clear algorithm educational materials separate for patients and parents, equipping providers with brief talking points in the EHR, and distilling information on data inputs to understandable explanations to improve provider communication efficacy [25]. Perspectives from youth in our sample emphasize the need for improved provider communication by connecting these efforts to the ability of youth to better practice personal autonomy and provide informed consent for next steps after risk identification. However, we also highlighted perspectives that risk models must ensure equity for youth with disabilities and those who are racially or systematically minoritized by balancing concerns for confidentiality with patient safety and allowing patients to opt out of the risk model, respectively. Additionally, challenges were presented in the preference of providers in our study for patients’ imminent risk status prior to upcoming visits. Ecological momentary assessments that sought to determine temporal variation in suicidality have found that suicidal ideation, suicide capability, and risk factors for suicidal ideation vary considerably, sometimes over the course of just a few hours [41,42]. To overcome this, suicide risk models typically predict the risk of a suicide attempt or death over the subsequent 30-90 days. Prognostic risk scores can include short-term and long-term risk factors to improve predictability [43] and better guide treatment plans over the following weeks to prevent future suicide [44]. Youth and caregiver participants of this study also stated support for using these tools to facilitate provider-patient discussions and identify treatment plans to prevent future suicide rather than focusing exclusively on youth patients at imminent risk. This challenge suggests the need to communicate model limitations to providers and carefully consider workflow implications to prevent implementation barriers related to increased burden.

### Limitations

The small sample sizes for both the quantitative survey and qualitative interview elements of these studies, along with the sole enrollment of providers and patients from the JHH or Kennedy Krieger Institute, present limitations related to generalizability and external validity. Additionally, assessing perspectives on implementing innovative technology in clinical care among providers at a renowned research institution may bias views toward greater reporting of acceptability or appropriateness. However, these providers may be better equipped to recognize the advantages and disadvantages of innovative technology, given their attunement to such developments. The time burden of participation and general interest in the study topic likely contributed to the small sample size, although this cannot be confirmed despite survey duration estimates provided. The limited representation of multiple caregivers or youth younger than 18 years in the patient and caregiver study may further limit generalizability. Finally, the codebook approach to qualitative analysis may have limited the ability to identify themes not within predefined frameworks such as the CFIR or the TFA [34], despite adding to the comparability of our findings to similar implementation science studies. The mixed methods study design and results synthesis by joint display sought to augment the limitations of the limited sample size.

Future qualitative work on the analysis of provider perspectives toward suicide risk model implementation efforts should recruit from a larger sample of multiple health care institutions to ensure the generalizability and external validity of these results. These studies should also prioritize exploring the perspectives of adolescent patients, their families, and health system leaders to determine whether, when, and how to implement ML approaches to risk identification. Importantly, perspectives from providers who reflect their patients’ cultures must be prioritized to inform culturally responsive and effective implementation. Recall bias may impact data collected assessing provider perspectives toward current suicide-related care, demonstrating the need for future implementation studies to evaluate real-time feedback from providers to better identify perceived barriers and facilitators. Despite these limitations, the agreement of the qualitative findings with recent similar research might speak to more broadly aligned perspectives in health systems across the United States.

### Conclusions

This mixed methods study contributes to the growing body of knowledge around the use of ML models on EHR data to identify patients at risk of suicide by exploring the perspectives of pediatric providers, adolescent and young adult patients, and caregivers on the implementation of these clinical tools. Health care providers in our sample were generally accepting of risk classification tools based on ML methods if there is trust in their accuracy and they are perceived to have added value. Building trust in the reliability and validity of the tool and empowering providers to act on risk flags were suggested to help effectively integrate such methods. Patients and caregiver participants were optimistic that these tools may facilitate discussions regarding mental health with providers and improve early detection and treatment efforts for at-risk youth. Acceptance of the implementation of these tools by patients and caregivers was contingent, however, on ensuring adequate data safety practices and encouraging patient autonomy through informed consent. The proposed implementation considerations from this study may support future implementation efforts to improve suicide prevention and care for youth.

## Supplementary material

10.2196/57602 Multimedia Appendix 1 Quantitative survey and qualitative interview guide for perspectives of pediatric providers, and qualitative interview guide for youth patient and caregiver perspectives on using machine learning methods to identify suicide risk in youth.

10.2196/57602 Multimedia Appendix 2 Quantitative survey results of pediatric provider perspectives on using machine learning methods to identify suicide risk in youth.

10.2196/57602 Multimedia Appendix 3 Joint display of the quantitative results, qualitative themes, and metainferences on barriers and facilitators to implementation of a youth suicide risk model, organized by Consolidated Framework for Implementation Research Framework Construct.

## Abbreviations

AI: artificial intelligence
CFIR: Consolidated Framework for Implementation Research
ED: emergency department
EHR: electronic health record
IRB: Institutional Review Board
JHH: Johns Hopkins Hospital
ML: machine learning
TFA: Theoretical Framework of Acceptability

## References

[1] Curtin SC, Garnett MF, Ahmad FB (2022). Provisional numbers and rates of suicide by month and demographic characteristics: United States, 2021.

[2] Chock MM, Bommersbach TJ, Geske JL, Bostwick JM (2015). Patterns of health care usage in the year before suicide: a population-based case-control study. Mayo Clin Proc.

[3] Stene-Larsen K, Reneflot A (2019). Contact with primary and mental health care prior to suicide: a systematic review of the literature from 2000 to 2017. Scand J Public Health.

[4] Ramchand R, Gordon JA, Pearson JL (2021). Trends in suicide rates by race and ethnicity in the United States. JAMA Netw Open.

[5] Yard E, Radhakrishnan L, Ballesteros MF (2021). Emergency department visits for suspected suicide attempts among persons aged 12–25 years before and during the COVID-19 pandemic—United States, January 2019–May 2021. MMWR Morb Mortal Wkly Rep.

[6] Ballard ED, Cwik M, Van Eck K (2017). Identification of at-risk youth by suicide screening in a pediatric emergency department. Prev Sci.

[7] Cwik MF, O’Keefe VM, Haroz EE (2020). Suicide in the pediatric population: screening, risk assessment and treatment. Int Rev Psychiatry.

[8] Aguinaldo LD, Sullivant S, Lanzillo EC (2021). Validation of the ask suicide-screening questions (ASQ) with youth in outpatient specialty and primary care clinics. Gen Hosp Psychiatry.

[9] Horowitz LM, Wharff EA, Mournet AM (2020). Validation and feasibility of the ASQ among pediatric medical and surgical inpatients. Hosp Pediatr.

[10] Haroz EE, Kitchen C, Nestadt PS, Wilcox HC, DeVylder JE, Kharrazi H (2021). Comparing the predictive value of screening to the use of electronic health record data for detecting future suicidal thoughts and behavior in an urban pediatric emergency department: a preliminary analysis. Suicide Life Threat Behav.

[11] Brent DA, Horowitz LM, Grupp-Phelan J (2023). Prediction of suicide attempts and suicide-related events among adolescents seen in emergency departments. JAMA Netw Open.

[12] DeVylder JE, Ryan TC, Cwik M (2019). Assessment of selective and universal screening for suicide risk in a pediatric emergency department. JAMA Netw Open.

[13] Franklin JC, Ribeiro JD, Fox KR (2017). Risk factors for suicidal thoughts and behaviors: a meta-analysis of 50 years of research. Psychol Bull.

[14] Walsh CG, Ribeiro JD, Franklin JC (2018). Predicting suicide attempts in adolescents with longitudinal clinical data and machine learning. J Child Psychol Psychiatry.

[15] Kusuma K, Larsen M, Quiroz JC (2022). The performance of machine learning models in predicting suicidal ideation, attempts, and deaths: a meta-analysis and systematic review. J Psychiatr Res.

[16] Belsher BE, Smolenski DJ, Pruitt LD (2019). Prediction models for suicide attempts and deaths: a systematic review and simulation. JAMA Psychiatry.

[17] Bentley KH, Zuromski KL, Fortgang RG (2022). Implementing machine learning models for suicide risk prediction in clinical practice: focus group study with hospital providers. JMIR Form Res.

[18] Yarborough BJH, Stumbo SP, Schneider JL, Richards JE, Hooker SA, Rossom RC (2022). Patient expectations of and experiences with a suicide risk identification algorithm in clinical practice. BMC Psychiatry.

[19] Reger MA, Ammerman BA, Carter SP (2021). Patient feedback on the use of predictive analytics for suicide prevention. Psychiatr Serv.

[20] Reale C, Novak LL, Robinson K (2020). User-centered design of a machine learning intervention for suicide risk prediction in a military setting. AMIA Annu Symp Proc.

[21] Haroz EE, Grubin F, Goklish N (2021). Designing a clinical decision support tool that leverages machine learning for suicide risk prediction: development study in partnership with Native American care providers. JMIR Public Health Surveill.

[22] Yarborough BJH, Stumbo SP (2023). A stakeholder-informed ethical framework to guide implementation of suicide risk prediction models derived from electronic health records. Arch Suicide Res.

[23] Richards JE, Yarborough BJH, Holden E (2022). Implementation of suicide risk estimation analytics to support mental health care for quality improvement. JAMA Netw Open.

[24] Yarborough BJH, Stumbo SP (2021). Patient perspectives on acceptability of, and implementation preferences for, use of electronic health records and machine learning to identify suicide risk. Gen Hosp Psychiatry.

[25] Davis M, Dysart GC, Doupnik SK (2024). Adolescent, parent, and provider perceptions of a predictive algorithm to identify adolescent suicide risk in primary care. Acad Pediatr.

[26] Lyon AR, Bruns EJ (2019). User-centered redesign of evidence-based psychosocial interventions to enhance implementation-hospitable soil or better seeds?. JAMA Psychiatry.

[27] Leech NL, Onwuegbuzie AJ (2009). A typology of mixed methods research designs. Qual Quant.

[28] Damschroder LJ, Aron DC, Keith RE, Kirsh SR, Alexander JA, Lowery JC (2009). Fostering implementation of health services research findings into practice: a consolidated framework for advancing implementation science. Implement Sci.

[29] Sekhon M, Cartwright M, Francis JJ (2017). Acceptability of healthcare interventions: an overview of reviews and development of a theoretical framework. BMC Health Serv Res.

[30] Posner K, Brown GK, Stanley B (2011). The Columbia-Suicide Severity Rating Scale: initial validity and internal consistency findings from three multisite studies with adolescents and adults. Am J Psychiatry.

[31] Kroenke K, Spitzer RL, Williams JBW (2001). The PHQ-9: validity of a brief depression severity measure. J Gen Intern Med.

[32] QUALTRICS AI + THE XM PLATFORM. Qualtrics XM.

[33] (2024). Dedoose version 9.2.12, cloud application for managing, analyzing, and presenting qualitative and mixed method research data. SocioCultural Research Consultants, LLC.

[34] Braun V, Clarke V (2023). Toward good practice in thematic analysis: avoiding common problems and be(com)ing a *knowing* researcher. Int J Transgend Health.

[35] King N, Symon G, Cassell C (1998). Qualitative Methods and Analysis in Organizational Research: A Practical Guide.

[36] Brooks J, King N (2014). Doing Template Analysis: Evaluating an End-of-Life Care Service Sage Research Methods Cases Part 1.

[37] Fetters MD, Curry LA, Creswell JW (2013). Achieving integration in mixed methods designs-principles and practices. Health Serv Res.

[38] Guetterman TC, Fetters MD, Creswell JW (2015). Integrating quantitative and qualitative results in health science mixed methods research through joint displays. Ann Fam Med.

[39] McCrudden MT, Marchand G, Schutz PA (2021). Joint displays for mixed methods research in psychology. Methods Psychol.

[40] Yarborough BJH, Stumbo SP, Schneider J, Richards JE, Hooker SA, Rossom R (2022). Clinical implementation of suicide risk prediction models in healthcare: a qualitative study. BMC Psychiatry.

[41] Bayliss LT, Hughes CD, Lamont-Mills A, du Plessis C (2024). Fluidity in capability: longitudinal assessments of suicide capability using ecological momentary assessments. Suicide Life Threat Behav.

[42] Kleiman EM, Turner BJ, Fedor S, Beale EE, Huffman JC, Nock MK (2017). Examination of real-time fluctuations in suicidal ideation and its risk factors: results from two ecological momentary assessment studies. J Abnorm Psychol.

[43] Simon GE, Johnson E, Lawrence JM (2018). Predicting suicide attempts and suicide deaths following outpatient visits using electronic health records. Am J Psychiatry.

[44] Penfold RB, Johnson E, Shortreed SM (2021). Predicting suicide attempts and suicide deaths among adolescents following outpatient visits. J Affect Disord.

