# Supplementary material for: Exploring the Perspectives of Pediatric Health Care Providers, Youth Patients, and Caregivers on Machine Learning Suicide Risk Classification: Mixed Methods Study
Source: J Med Internet Res. 2025 Aug 19;27:e57602. doi: 10.2196/57602 (PMC12364417; doi:10.2196/57602)
Supplement: Multimedia Appendix 1 [file jmir-v27-e57602-s001.docx]

**Interview Guide for Providers**

Introduction

Thank you for speaking with me today. Our conversation today will be about how providers (primary care/ED) can better identify pediatric patients who might be at risk for suicide and how to best support them and their families.

We would like to hear your thoughts on using a risk classification tool that uses machine learning on electronic health records data [such as patient screening responses and mental health history] to classify adolescents into suicide risk categories. We would want to know about the tool itself, as well as, gather information about the corresponding care and workflow.

This interview will be audio-recorded and professionally transcribed. Your answers will be kept confidential and all personal information will be de-identified. The recording will be destroyed after it is transcribed and analyzed. Please note, you do not have to answer every question and you can end the interview at any time. Before we begin, do you have any questions? Do I have your consent to record this interview?

Part 1: Exploratory Inquiry

These first few questions will be about your thoughts on suicide care more generally.

1. How do you feel about current suicide prevention programs/practices/processes and assessment tools that are available related to the intervention? Probes: to what extent do current programs fail to meet existing needs? How might this tool address these needs? – *(code: tension for change)*
2. What role do you feel that [insert provider type here] providers should play in adolescent suicide prevention? – *(code: perceived need)*
3. Currently, how comfortable do you feel about talking with patients and their families about suicide? Probes: For example, giving families anticipatory guidance or talking about general risk factors for suicide at a patient’s regular check-up? How comfortable would you feel discussing with families how to handle risk for a patient who endorses suicide or is flagged as high risk?
4. How do you currently use health record and/or screening/risk assessment data to identify adolescent patients who may be at risk for suicide? Probes: How do you use clinical information from patient assessments to identify adolescents who may be at risk for suicide? How do you use screeners [e.g. ASQ/PHQ/CSSR-S] to identify adolescent patients who may be at risk for suicide?

Part 2: Reactions to prototype and perspectives on implementation


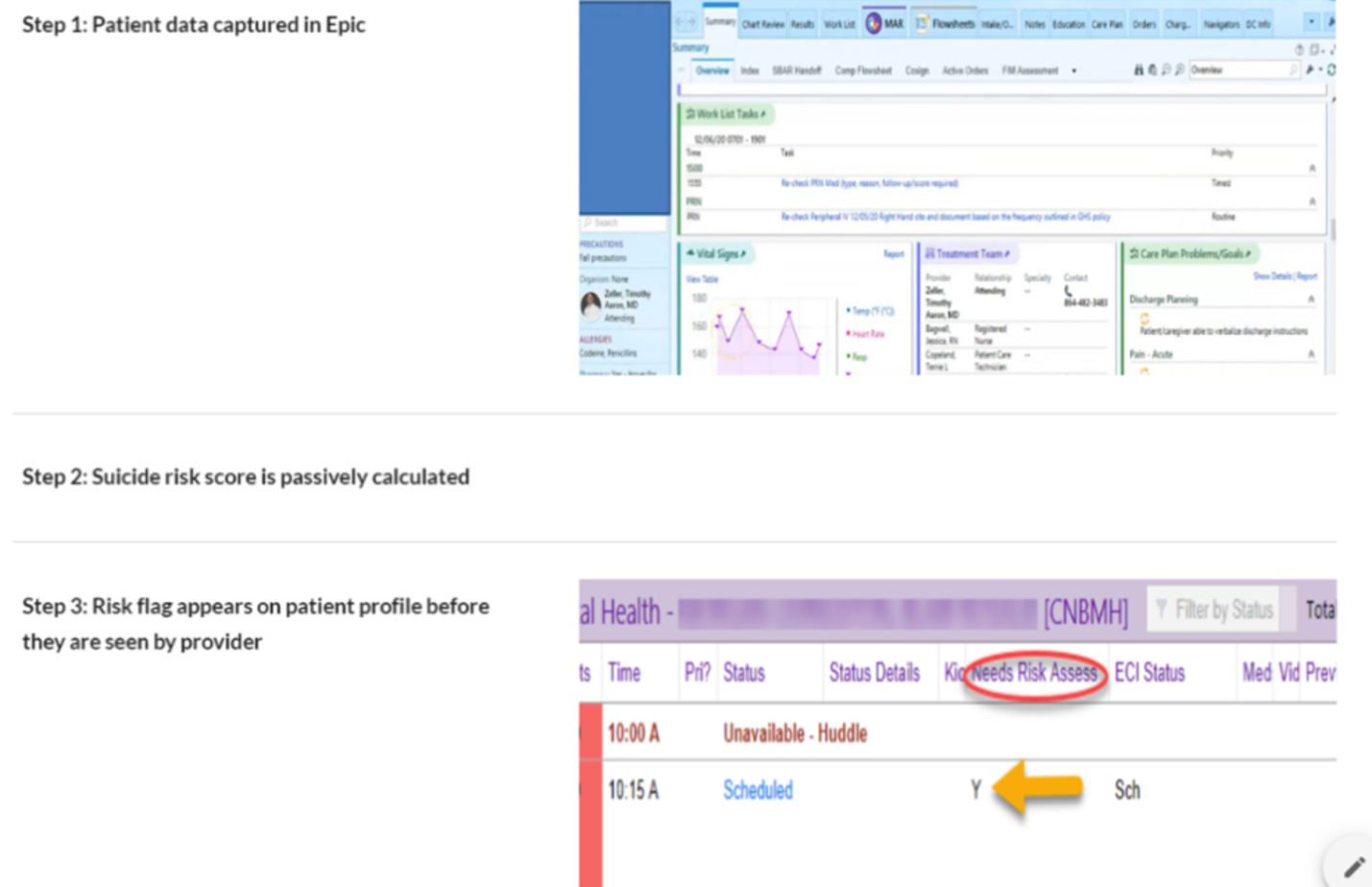


This is an example of what a suicide identification tool in the EHR might look like. [walk through steps]. We would like to hear your thoughts, insights, suggestions, and perceived challenges in order to improve the tool and/or its use in your work setting. Please take a few minutes to look at it and let me know when you are ready.

1. How do you use risk identification models or calculators (e.g. Framingham Risk Score; risk of readmission, sepsis risk, etc.) in your current work? Probes: When do they inform your care? And How? What do you think about these approaches to help identify people at high risk of suicide?

1. What are your initial reactions to this sample prototype?

1. How would you feel about using a tool like this one to guide you in identifying and assessing potential suicide risk in your patients? Probes: would you be comfortable getting a flag in EHR if a patient was at risk for suicide?

1. How do you envision the use of a suicide risk classification tool in practice? Probes: Would you integrate it during the other standard of care screenings you do? Would you ask the parent/caregiver to step out? Would you ask your patients directly or have them fill out the questionnaire on their own?

1. How complicated do you think this tool is? Do you have suggestions on how to improve it? Probes: please consider the following aspects of the intervention: duration, scope, intricacy and number of steps involved and whether the intervention reflects a clear departure from previous practices. – *(code: complexity)*
2. If a patient is flagged as high risk by the tool, when and how would you want to be notified? Probes: is it helpful to know a patient's risk status before you meet with them for an appointment (primary care/outpatient care only)? Before you discharge them? – *(code: design quality & packaging)*
3. How might this tool affect your clinical workflow? – *(code: networks & communications)*
4. Who should be responsible for communicating results to patients and their families? - *(code: networks and communications)*
5. What (additional) supports, such as online resources, training, ongoing support, marketing materials, or a toolkit, would be helpful in implementing and using this tool and talking about suicide risk with patients and their families? – *(code: design quality & packaging)*
6. How do you think adolescent patients and their families served by your organization would respond to the use of this tool? – *(code: patient needs & resources)*
7. If you had a patient who is flagged as high risk by the tool, how comfortable do you feel with conveying this information to the patient and their family? Please describe what you would say to convey this information to the patient? To their family? – *(code: conveying information to patients & their families)*
8. Are there any special considerations or adaptations needed to inform implementation of a tool like this with adolescents who belong to specific populations? Probes: Black, Hispanic, Indigenous populations, adolescents with disabilities, youth involved in the child welfare or criminal justice systems? – *(code: patient needs & resources)*

Part 3: Perspectives on implementation

1. Compared to what you do now, how helpful do you think implementing this tool will be? Please tell us your thoughts on the need for such a tool? – *(codes: relative priority, tension for change)*
2. What barriers (if any) do you anticipate coming across in implementing a suicide risk classification tool?
3. Do you have any ethical concerns with the use of this tool? Why? Probes: How do the ethical considerations of this tool differ from other existing risk calculators that use patient data, such as risk calculators for cancer or heart disease? - *(code: ethicality)*
4. What suggestions or ideas do you have to effectively and efficiently implement a suicide risk classification tool in your work?
5. Is there anything else you would want to share with us to help inform the implementation of risk models for suicide?

Thank you so much for taking the time to talk with me today. Please feel free to reach out if you have any additional questions or thoughts that come up following today’s interview. On behalf of the entire research team, thank you for your contribution to this important work.

**Provider Survey**

Intro

Thank you for your interest in participating in this survey. We would like to hear your thoughts on using a tool that uses machine learning on electronic health records data to classify adolescents into suicide risk categories (e.g., low risk, medium risk, high risk). Machine learning based classification algorithms have been shown to increase our ability to identify individuals at risk. However, we also know that suicide risk is rare, so there will be times when any tool, will falsely identify people at risk or miss people who may indeed be at risk. We are trying to understand how best to use this type of tool, as well as, gather information about the corresponding care and workflow.

This survey will take approximately 20 to 30 minutes to complete. Are you interested in hearing more?

Yes

No

(If yes) <Online Consent Form if Applicable>

(If no) End survey

—---------------------------------------------------------page break—-------------------------------------------------------------

Please read the following vignette carefully.

Joe is a 16 year old male who has been seeing the same doctor for his depression for the past 3 years. He takes his doctor’s advice to manage any symptoms he has. The hospital he goes to is using a new system that calculates the risk of some medical conditions using the information stored in the electronic medical record from his past visits to the hospital. The system identifies that Joe is at high risk of suicide.

The next section will ask what you think about how to use this type of tool and the possible barriers and facilitators to its use.

—---------------------------------------------------------page break—-------------------------------------------------------------

1. How would Joe’s risk status be most useful for informing care? (select all that apply)
2. To inform primary care visits
3. To inform ER care
4. To inform discharge plans
5. Other (Please describe________)

1. When would you like to see Joe’s risk status (select all that apply)?
2. Immediate notification to your email and/or in basket
3. Best Practice Alert
4. Notification on Joe’s chart prior to seeing him for an upcoming visit
5. Notification in the chart or otherwise after seeing Joe but before he is discharged or the visit ends
6. Other (Please explain where and when you would want to see this information)

1. What is the most important use of this type of risk flag?
2. To inform care for imminent risk
3. To inform care for risk that may occur in the next 30 days
4. To inform care for risk that may occur in the next 6 months
5. To inform care for risk that may occur over the next year

1. How would you want this tool to integrate with your existing processes?
2. Use it instead of a manual screening tool
3. Use it in conjunction with a manual screening tool
4. Not use it at all
5. Other (please describe)

1. How much would you alter care for someone classified as medium risk vs. high risk of suicide attempt or death?
2. *Slider tool here - with 0 meaning no change to care between the two, and 10 meaning a lot of differences in care*
3. *Please describe your rationale for your response, and include what you would do with this information in your workflow*

1. How would you want Joe’s risk status to be conveyed to him?
2. Automatic notification to Joe and/or his guardian via mychart
3. Social worker/Nurse/Aid calling Joe and/or his guardian to notify immediately
4. Schedule a separate in-person visit to convey this information to Joe
5. Discuss during upcoming consult or visit with Joe
6. Other?

—---------------------------------------------------------page break—-------------------------------------------------------------

The next few questions ask about your opinions about using this type of tool. (1-5, strongly disagree to strongly agree)

1. I think I would use this type of suicidal risk identification tool in my work.
2. I would find this type of suicidal risk identification tool unnecessarily complex
3. It seems possible to implement the suicidal risk identification tool where I work.
4. I think his type of suicidal risk identification tool would help our current suicide identification or risk assessment processes.
5. I trust the evidence that supports the development and implementation of the suicidal risk identification tool
6. I think this type of tool would improve the care I provide to my patients.

Now we would like to ask you some questions about the implementation context for this type of tool. Please answer the following questions as best you can

| **Item** | Indicate your agreement with this statement: |  | What is the likely effect of this barrier / facilitator on your ability to implement the classification tool? |
| --- | --- | --- | --- |
|  | **1 - DISAGREE:** This means the item is a potential barrier | This barrier will have | 0 - Weak/no effect  1 - Strong effect |
|  | **2 - Neutral** |  |  |
|  | **3 - AGREE:** This means the item is a potential facilitator | This facilitator will have | 0 - Weak/no effect  1 - Strong effect |
| Using a suicide classification tool in the EHR will make identification of patients at risk of suicide less complicated (*complexity)* |  |  |  |
| This type of tool would be too costly for the health system (*Cost)* |  |  |  |
| There is a need in my work for different suicide prevention programs/practices/processes and assessment tools (*Tension for change)* |  |  |  |
| Implementing a suicide classification tool like this is aligned with the goals of my workplace leadership (*Relative priority)* |  |  |  |
| Having this tool will improve communication with other providers on the patients care team *(networks & communications*) |  |  |  |
| Having this tool will make it easier to communicate the results of a suicide risk flag to patients and their families *(networks & communications*) |  |  |  |
| I have concerns over the potential ethical implications of using a tool like this (*ethics)* |  |  |  |
| If you have ethical concerns, please describe |  |  |  |

**Interview Guide for Patients and Caregivers**

Introduction

Thank you for speaking with me today. We will talk about ways that doctors could be able to tell if a young person might be at risk for suicide and how to support them and their families.

We would like to hear your thoughts on a new tool that could be used in hospitals. This tool looks at things a person has done in the past for their health, such as how many times they visited the hospital and why, to see if they might pass away by suicide within the next year.

Part 1: Exploratory Inquiry

1. Have you heard of artificial intelligence? [probe for understanding] What are your thoughts on it being used to help doctors know who is sick?
2. What about doctors using it to help young people?
3. What about it being used for physical health versus mental health?

Part 2: Vignette-based Inquiry

I will provide you with a story to read. Please follow along as I read it aloud.

Trey is a 16 year old who has been seeing the same doctor for his depression and anxiety for the past 3 years. He takes his doctor’s advice to manage his symptoms. While on vacation, Trey accidentally twists his ankle and must go to the emergency room. The hospital he visits is using a tool that tries to tell if a patient may attempt suicide or may hurt themselves based on the information like how many times they have gone to the hospital and for what. The emergency room doctor sees that the tool says that Trey might be in danger of attempting suicide within the next year.

1. How do you feel about the tool that used Trey’s medical information to see if he might attempt suicide within the next year?  – *(code: affective attitude)*

1. What are the good things about a doctor using this tool to find out if a patient might be at risk of suicide? Probes: What are the downsides? Why? –  *(code: Opportunity Costs)*
2. Could you think of any problems that might happen if this type of tool was used in hospitals? Are there any ethical or privacy concerns? – *(code: ethicality)*

1. Who at the hospital should be able to see that Trey might attempt suicide?

1. What do you think should happen after the emergency room doctor sees that the tool predicts that Trey might attempt suicide? What should not happen? – *(code: suggestions/preferences, affective attitude)*

1. How should Trey be told that the tool predicted that he might attempt suicide?
2. Who should tell him about that? Who should not?
3. Would it be okay for the nurse to tell him or should it be his doctor?
4. Should he be told by a mental health doctor only?

1. What do you think Trey would want to know including the prediction that the tool made?

1. Should Trey have to provide his consent/agree for his health information to be used in this type of tool? Why or why not? – *(code: ethicality)*

1. How effective would this tool be in helping Trey avoid harming himself in the future? – *(code: perceived effectiveness)*

1. If someone your age/a young person is thinking about suicide, what do you think is important for doctors to know about how they want to be treated? – *(code: affective attitude)*

1. A lot of hospitals use paper and pencil tests or they ask patients directly if they have thought about suicide. The tool I described would only use the information that the hospital already has, such as how many times Trey went to hospital this past year. By using the tool, a doctor would not ask the patient directly about suicide. What are your thoughts on the advantages and disadvantages of these types of tools?

1. How would you feel if private information, such as sexual orientation/what kinds of people a person likes, were used for predicting their risk of suicide attempt? – *(code: affective attitude)*

1. Overall, what are your thoughts about this type of tool and whether it would be helpful?

Thank you very much for your participation. In case you would like to talk to someone about your mental health, I have some resources. You can call the 988 Suicide & Crisis Lifeline or the SAMHSA National Helpline by calling 1-800-622-HELP (4357). They both offer free and confidential support and are available 24/7. You can also text HOME to 741741 to connect with a Crisis Counselor. This is also free and available 24/7. [probe if they would like this information sent in the chat]. If you have any other thoughts, questions, or concerns about this study, please reach out to us. [end call]
