# Supplementary material for: Exploring the Perspectives of Pediatric Health Care Providers, Youth Patients, and Caregivers on Machine Learning Suicide Risk Classification: Mixed Methods Study
Source: J Med Internet Res. 2025 Aug 19;27:e57602. doi: 10.2196/57602 (PMC12364417; doi:10.2196/57602)
Supplement: Multimedia Appendix 2 [file jmir-v27-e57602-s002.docx]

**Table** **S1.** Provider perspectives towards youth suicide risk models among those who answered one or more questions on the quantitative survey (N = 45).

|  | **Psychologist, N = 23** | **Physician,**  **N = 16** | **Social Worker,**  **N = 3** | **Nurse or Nurse Practitioner, N = 3** | **Total,**  **N = 45** |
| --- | --- | --- | --- | --- | --- |
| *Preferred usage of risk score,* n (%)^1^ |  |  |  |  |  |
| Primary Care | 17 (74) | 12 (75) | 3 (100) | 2 (67) | 34 (76) |
| Emergency Care | 5 (22) | 10 (63) | 2 (67) | 2 (67) | 19 (42) |
| Discharge Plan | 14 (61) | 11 (69) | 2 (67) | 3 (100) | 30 (67) |
| Other | 4 (17) | 2 (13) | 1 (33) | 1 (33) | 8 (18) |
| *Preferred time of risk notification*,  n (%)^2^ |  |  |  |  |  |
| Immediately upon calculation | 2 (9) | 4 (25) | 1 (33) | 0 (0) | 7 (16) |
| Prior to upcoming visit | 19 (82) | 12 (75) | 2 (67) | 3 (100) | 36 (80) |
| Before discharge/end of visit | 2 (9) | 0 (0) | 0 (0) | 0 (0) | 2 (4) |
| *Preferred risk model outcome to inform care*, n (%)^3^ |  |  |  |  |  |
| Current suicide risk (imminent risk) | 14 (61) | 9 (56) | 2 (67) | 2 (67) | 27 (60) |
| 30-day suicide risk | 6 (26) | 3 (19) | 0 (0) | 0 (0) | 9 (20) |
| 6-month suicide risk | 1 (4) | 2 (13) | 0 (0) | 1 (33) | 4 (9) |
| 12-month suicide risk | 2 (9) | 2 (13) | 1 (33) | 0 (0) | 5 (11) |
| *Preferred use of model predicted risk*, n (%)^5^ |  |  |  |  |  |
| Replace manual screening tool | 1 (4) | 2 (13) | 0 (0) | 2 (67) | 5 (11) |
| In conjunction with manual screening tool | 17 (74) | 11 (69) | 2 (67) | 0 (0) | 30 (67) |
| Not use it at all | 3 (13) | 3 (19) | 0 (0) | 1 (33) | 7 (15) |
| Other | 2 (9) | 0 (0) | 1 (33) | 0 (0) | 3 (7) |
| *Degree of altering care for someone classified at medium risk vs high risk of suicide attempt/death on scale of 1-10*^6^ |  |  |  |  |  |
| Average score, mean (SD) | 6.0 (2.3) | 6.1 (2.9) | 6.3 (0.6) | 6.3 (3.2) | 6.0 (2.4)^6^ |
| Missing Responses | 0 | 1 | 0 | 0 | 1 |
| *Preferred communication of risk status to patient,* n (%)^7^ |  |  |  |  |  |
| Automated notification to patient and/or guardian via EHR | 0 (0) | 0 (0) | 0 (0) | 0 (0) | 0 (0) |
| Patient and/or guardian notification by clinical staff (SW, RN, aid) | 4 (17) | 1 (6) | 1 (33) | 0 (0) | 6 (13) |
| During separate in-person visit | 4 (17) | 5 (31) | 1 (33) | 0 (0) | 10 (22) |
| During upcoming visit | 9 (39) | 7 (44) | 0 | 2 (67) | 18 (40) |
| Other | 6 (26) | 3 (19) | 1 (33) | 1 (33) | 11 (24) |
| ^1^ After reading the vignette about a hypothetical patient named “Joe” (provided in Supplemental File 1), participants were asked, “How would Joe’s risk status be most useful for informing care?” It should be noted that participants were allowed to select multiple responses.  ^2^ To assess preferred time of notification, participants were asked “When would you like to see Joe’s risk status?”  ^3^ To assess preferred outcome of the risk score, participants were asked “What is the most important use of this type of risk flag?”  ^4^ To assess preferred use of the model risk score, participants were asked “How would you want this tool to integrate with your existing processes?” Providers who selected other stated 1) it depends on likelihood of false positives/false negatives, 2) during clinical interview, or 3) only for new patients.  ^5,6^ SD = Standard Deviation; Participants were asked to rate on a scale of 1-10 “How much would you alter care for someone classified as medium risk vs. high risk of suicide attempt or death?” The mean and standard deviation by occupation type was reported. It should be noted that one physician did not answer this question. The mean and standard deviation provided in the total column is the overall score disaggregated by participant occupation.  ^7^To assess preferred communication of risk status, participants were asked “How would you want Joe’s risk status to be conveyed to him?” Providers who selected other stated 1) it depends on the risk status, 2) during current visit/immediately, 3) not immediately documented. | | | | | |

**Table S2.** Provider perspectives towards the usability of a potential suicide risk identification model assessed through adapted usability scale among those who answered at least one of the following questions (N = 44).

|  | **Psychologist, N = 23** | **Physician,**  **N = 16^1^** | **Social Worker,**  **N = 3** | **Nurse or Nurse Practitioner, N = 3** | **Total,**  **N = 45** |
| --- | --- | --- | --- | --- | --- |
| *Individual Items^2^,* mean (SD) |  |  |  |  |  |
| 1. I think I would use this type of suicidal risk identification tool in my work. | 3.7 (1.0) | 3.4 (1.0) | 4.3 (0.6) | 2.7 (1.5) | 3.5 (1.0) |
| 2. I would find this type of suicidal risk identification tool unnecessarily complex^3^ | 2.7 (1.1) | 2.7 (0.9) | 3.7 (1.5) | 2.0 (1) | 2.8 (1.1) |
| 3. It seems possible to implement the suicidal risk identification tool where I work. | 3.8 (0.9) | 3.4 (0.8) | 4.0 (1) | 4.0 (1.4) | 3.7 (0.9) |
| 4. I think this type of suicidal risk identification tool would help our current suicide identification or risk assessment processes. | 3.3 (0.9) | 3.4 (1.1) | 4.3 (0.6) | 3.0 (1.7) | 3.4 (1.0) |
| 5. I trust the evidence that supports the development and implementation of the suicidal risk identification tool | 3.2 (1.0) | 3.0 (0.8) | 4.0 (0) | 3.7 (1.2) | 3.2 (0.9) |
| 6. I think this type of tool would improve the care I provide to my patients. | 3.2 (1.1) | 3.3 (1.0) | 4.0 (0) | 3.3 (0.6) | 3.3 (1.0) |
| *Overall score,^4^* mean (SD) | 3.4 (0.8) | 3.3 (0.8) | 3.8 (0.2) | 3.4 (0.1) | 3.4 (0.7)^3^ |
| ^1^ One missing physician provider response was omitted from all item scores for physicians and from the total score.  ^2^ SD = Standard deviation; The Adapted Intervention Usability Scale asked participants to rate their opinions about using this type of suicide risk tool. Responses ranged from “strongly disagree” to “strongly agree” and were scored on a scale of 1 to 5. The mean and standard deviation were reported. For all items besides 2, a mean item score of 3 equates to neither agreement nor disagreement, a mean item score above 3 equates to overall agreement, and a mean item score below 3 equates to overall disagreement. Item score averages were given by occupation type and were disaggregated by occupation type in the total column.  ^3^ It should be noted that the item 2 score was not reverse coded  ^4^ To calculate the overall score, item 2 was reverse coded, and item scores were summed for each provider. Total score averages were given by occupation type and were disaggregated by occupation type in the total column. | | | | | |

**Table S3.** CFIR potential facilitators and barriers, and their likely reported effects by occupation type.

|  | **Psychologist, N = 23** | **Physician,**  **N = 16** | **Social Worker**  **N = 3** | **Nurse or Nurse Practitioner, N = 3** | **Total,**  **N = 45^1^** |
| --- | --- | --- | --- | --- | --- |
| *CFIR Code: Complexity* | | | | | |
| 1. Using a suicide classification tool in the EHR will make identification of patients at risk of suicide less complicated, n (%) |  |  |  |  |  |
| Facilitator | 8 (35) | 4 (25) | 1 (33) | 1 (33) | 14 (31) |
| Barrier | 5 (22) | 4 (25) | 0 (0) | 0 (0) | 9 (20) |
| Neutral | 8 (35) | 5 (31) | 2 (67) | 0 (0) | 15 (33) |
| Missing | 2 (9) | 3 (19) | 0 (0) | 2 (67) | 7 (16) |
| *CFIR Code Cost* | | | | | |
| 2. This type of tool would not be too costly for the health system, n (%)^2^ |  |  |  |  |  |
| Facilitator | 14 (61) | 4 (25) | 1 (33) | 0 (0) | 19 (42) |
| Barrier | 0 (0) | 1 (6) | 0 (0) | 0 (0) | 1 (2) |
| Neutral | 7 (30) | 8 (50) | 2 (67) | 1 (33) | 18 (40) |
| Missing | 2 (9) | 3 (19) | 0 (0) | 2 (67) | 7 (16) |
| *CFIR CODE Tension for Change* | | | | | |
| 3. There is a need in my work for different suicide prevention programs/practices/processes and assessment tools, n (%) |  |  |  |  |  |
| Facilitator | 10 (43) | 5 (31) | 3 (100) | 0 (0) | 18 (40) |
| Barrier | 7 (30) | 4 (25) | 0 (0) | 0 (0) | 11 (24) |
| Neutral | 4 (17) | 4 (25) | 0 (0) | 1 (33) | 9 (20) |
| Missing | 2 (9) | 3 (19) | 0 (0) | 2 (67) | 7 (16) |
| *CFIR Code Networks & Communications* | | | | | |
| 4. Implementing a suicide classification tool like this is aligned with the goals of my workplace leadership, n (%) |  |  |  |  |  |
| Facilitator | 14 (61) | 7 (44) | 1 (33) | 1 (33) | 23 (51) |
| Barrier | 1 (4) | 2 (13) | 0 (0) | 0 (0) | 3 (7) |
| Neutral | 6 (26) | 4 (25) | 2 (67) | 0 (0) | 12 (27) |
| Missing | 2 (9) | 3 (19) | 0 (0) | 2 (67) | 7 (16) |
| 5. Having this tool will improve communication with other providers on the patients care team, n (%) |  |  |  |  |  |
| Facilitator | 13 (57) | 7 (44) | 3 (100) | 1 (33) | 24 (53) |
| Barrier | 3 (13) | 2 (13) | 0 (0) | 0 (0) | 5 (11) |
| Neutral | 5 (22) | 4 (25) | 0 (0) | 0 (0) | 9 (20) |
| Missing | 2 (9) | 3 (19) | 0 (0) | 2 (67) | 7 (16) |
| 6. Having this tool will make it easier to communicate the results of a suicide risk flag to patients and their families, n (%) |  |  |  |  |  |
| Facilitator | 8 (35) | 5 (31) | 2 (67) | 0 (0) | 15 (33) |
| Barrier | 5 (22) | 1 (6) | 0 (0) | 1 (33) | 7 (16) |
| Neutral | 8 (35) | 7 (44) | 1 (33) | 0 (0) | 16 (36) |
| Missing | 2 (9) | 3 (19) | 0 (0) | 2 (67) | 7 (16) |
| *CFIR Code Ethics* | | | | | |
| 7. I do not have concerns over the potential ethical implications of using a tool like this, n (%) |  |  |  |  |  |
| Facilitator | 2 (9) | 1 (6) | 0 (0) | 1 (33) | 4 (9) |
| Barrier | 14 (61) | 7 (44) | 1 (33) | 0 (0) | 22 (49) |
| Neutral | 5 (22) | 5 (31) | 2 (67) | 0 (0) | 12 (27) |
| Missing | 2 (9) | 3 (19) | 0 (0) | 2 (67) | 7 (16) |
| ^1^ There were 45 providers in total who responded to at least one of the survey questions, but only N = 38 responded to at least one of the CFIR questions. More specifically, there were 21 psychologists, 13 physicians, 3 social workers, and 1 nurse practitioner who completed at least one of these questions. To reduce bias, we reported all survey results in terms of the overall sample size of 45 providers who completed at least one question.  ^2^ It should be noted that the original wording of this question stated, “This type of tool would be too costly for the health system.” This question was reworded, and responses were reverse coded for consistency with all statements in this table (i.e. if providers disagreed with the statement, they viewed cost as a barrier).  ^3^ It should also be noted that the original wording of the statement said, “I have concerns over the potential ethical implications of using a tool like this.” The question was re-worded in the table and responses were recoded to be consistent with all statements in the table (i.e. if providers disagreed with the statement, they foresaw ethical barriers to implementations). | | | | | |
